# Predicting haemodynamic networks using electrophysiology: The role of non-linear and cross-frequency interactions

**DOI:** 10.1016/j.neuroimage.2016.01.053

**Published:** 2016-04-15

**Authors:** P. Tewarie, M.G. Bright, A. Hillebrand, S.E. Robson, L.E. Gascoyne, P.G. Morris, J. Meier, P. Van Mieghem, M.J. Brookes

**Affiliations:** a Sir Peter Mansfield Magnetic Resonance Centre, School of Physics and Astronomy, University of Nottingham, Nottingham, UK; b Department of Clinical Neurophysiology and MEG Center, VU University Medical Centre, Amsterdam, The Netherlands; c Delft University of Technology, Faculty of Electrical Engineering, Mathematics and Computer Science, Delft, The Netherlands

**Keywords:** Magnetoencephalography, MEG, Functional magnetic resonance imaging, fMRI, Functional connectivity, Resting state network, RSN, Relationship between fMRI and MEG, Mapping, Multivariate Taylor series

## Abstract

Understanding the electrophysiological basis of resting state networks (RSNs) in the human brain is a critical step towards elucidating how inter-areal connectivity supports healthy brain function. In recent years, the relationship between RSNs (typically measured using haemodynamic signals) and electrophysiology has been explored using functional Magnetic Resonance Imaging (fMRI) and magnetoencephalography (MEG). Significant progress has been made, with similar spatial structure observable in both modalities. However, there is a pressing need to understand this relationship beyond simple visual similarity of RSN patterns. Here, we introduce a mathematical model to predict fMRI-based RSNs using MEG. Our unique model, based upon a multivariate Taylor series, incorporates both phase and amplitude based MEG connectivity metrics, as well as linear and non-linear interactions within and between neural oscillations measured in multiple frequency bands. We show that including non-linear interactions, multiple frequency bands and cross-frequency terms significantly improves fMRI network prediction. This shows that fMRI connectivity is not only the result of direct electrophysiological connections, but is also driven by the overlap of connectivity profiles between separate regions. Our results indicate that a complete understanding of the electrophysiological basis of RSNs goes beyond simple frequency-specific analysis, and further exploration of non-linear and cross-frequency interactions will shed new light on distributed network connectivity, and its perturbation in pathology.

## Highlights

• We introduce a mathematical model to predict fMRI-based RSNs using MEG.
• Our model is based on a multi-variate Taylor series expansion.
• The electrophysiological basis of RSNs goes beyond frequency-band specific analysis.
• RSNs result 1) from multiple frequency bands and cross-frequency coupling.
• RSNs result 2) from direct and shared electrophysiological connectivity.

## Introduction

Functional neuroimaging has brought about a revolution in neuroscience following the discovery of spatio-temporal patterns in measurable (resting and task positive) brain “activity” (Deco et al., 2011, Engel et al., 2001). In this context, functional Magnetic Resonance Imaging (fMRI) has been the dominant imaging modality and has provided the neuroscience community with a wealth of information about brain networks and the functional connectivities that define them (Bullmore and Sporns, 2009). However, given the limited temporal resolution and the indirect assessment of neuronal activity with fMRI, research groups are increasingly beginning to employ magnetoencephalography (MEG), either alone or alongside fMRI, to better characterise patterns of functional connectivity (Larson-Prior et al., 2013). MEG offers specific advantages for network characterisation, including (i) more direct assessment of electrophysiological activity and (ii) excellent (millisecond) temporal resolution. These advantages suggest that the role of MEG in network characterisation will become even more prominent, particularly given the increasing interest in the dynamics of functional connectivity (Baker et al., 2014, Hutchison et al., 2013, O'Neill et al., 2015). However, despite excellent promise, the relationship between functional networks obtained from haemodynamic and electrophysiological measurements remains poorly understood and in order to reach the full potential of multimodal studies there is a pressing need for a quantitative framework that better elucidates this relationship.

Initial studies on the relationship between MEG and fMRI measured functional connectivity have highlighted a degree of spatial overlap between networks reconstructed independently from these two modalities (Brookes et al., 2011a, de Pasquale et al., 2010, de Pasquale et al., 2012, Hipp et al., 2012). This spatial overlap extended to the well-known independent component analysis (ICA) obtained resting state networks (RSNs) (Brookes et al., 2012a, Brookes et al., 2011b, Hall et al., 2013, Luckhoo et al., 2012) and to parcellation based whole brain functional connectivity (Liljeström et al., 2015, Tewarie et al., 2014). The observed similarity between RSNs measured using the two modalities is compelling and extends the relationship between haemodynamics and electrophysiology that has been observed previously in task based studies (Brookes et al., 2005, Logothetis, 2003, Menon et al., 1997, Mullinger et al., 2013, Musso et al., 2010, Singh, 2012, Singh et al., 2002, Stevenson et al., 2012). This said, there are significant limitations to the previous approaches. Firstly, whilst most studies were based upon measurements of neural oscillations (rhythmic electrophysiological activity in large scale cell assemblies), many studies probed individual frequency bands in isolation (e.g. alpha, beta etc.), without reference to a bigger ‘pan-spectral’ picture. In fact, rather than reflecting a single frequency band, fMRI networks more likely result from an amalgam of electrophysiological connectivity across all frequency bands (Hipp and Siegel, 2015). Secondly, the rich nature of the electrophysiological signal facilitates multiple independent measurements of functional connectivity (Schölvinck et al., 2013). For example some studies (Stam et al., 2007) look for a phase relationship (e.g. phase synchronization) between signals from separate regions; others look for correlation between the amplitude envelopes of oscillations (Brookes et al., 2011a). These separate mechanisms of interaction have been described as independent and intrinsic modes of coupling in the brain (Engel et al., 2013). However, for comparison with fMRI, they are typically treated in isolation whereas haemodynamic functional connectivity is likely to be derived from a combination of these modes. In addition, most studies employ only a simple visual inspection of network patterns (Brookes et al., 2011a, Brookes et al., 2011b, Hipp et al., 2012), and no studies have yet tested for non-linear interactions between MEG derived measurements and fMRI. It follows therefore that a single framework enabling (i) integration of electrophysiological data from multiple frequency bands, (ii) integration of multiple metrics of functional connectivity and (iii) the combination of both linear and non-linear interactions within and between MEG frequency bands and metrics, would represent a powerful step forward in understanding the relationship between haemodynamic and electrophysiological functional networks.

In the present study, we introduce a framework to characterise the potentially multivariate, (non)-linear relationships between MEG and fMRI obtained functional networks. Our method is based upon the assumption that the relationship between MEG and fMRI can be translated to a multi-dimensional mathematical function, which can be approximated using a multivariate Taylor series (Van Mieghem, 2010). It is noteworthy that an approach conceptually similar to this (although univariate) has been applied successfully to the relationship between structural and functional networks (Meier et al., 2016). Here we use a multivariate Taylor expansion to investigate the relationship between fMRI and MEG networks. This expansion allows us not only to integrate network estimates for different frequency bands in a linear and non-linear combination, but also to question the extent to which each frequency-specific MEG network explains observable fMRI network structure. In addition, since a multivariate Taylor expansion also contains cross-terms, the contribution of cross-frequency coupling to the measured fMRI networks can also be probed.

## Theory

It is well known that a truncation of Taylor series can be used as an approximation of a function around a development point. In general, Taylor series are evaluated for known functions, the accuracy of the expansion being critically dependent on the number of terms used. This can be quantified by a reduction in the error between the function itself and the truncated expansion. In the case of a mapping between MEG and fMRI, the function itself is unknown and therefore our Taylor coefficients are also unknown. However, if the function between MEG and fMRI is analytical around a development point, we can use a Taylor series, even in the absence of a known function, because we are able to estimate Taylor coefficients for every term using non-linear least-squared fitting methods.

We consider MEG derived connectivity matrices **W**_*f*_, where *f* refers to frequency band (1 = delta, 2 = theta, 3 = alpha, 4 = beta, 5 = gamma), and the fMRI connectivity matrix **V**. The matrices **W**_*f*_ (for all *f*) and **V** are symmetric weighted adjacency matrices, where the elements can take real values between [− 1, 1]. The **W**_*f*_ matrices can be grouped together, for which we write **W** = (**W**_1,_**W**_2,_, **W**_3,_, **W**_4,_, **W**_5,_). Every element corresponds to a functional connectivity value between two brain regions in some specific frequency band, where regions are defined by a parcellation atlas (Tzourio-Mazoyer et al., 2002). We assume that there is a dependency between MEG and fMRI connectivity matrices, which implies that there is a function,

(1) $$V=F\left(W\right)$$

which maps MEG connectivity matrices *W* onto an fMRI connectivity matrix *V*. If we assume that this function is analytical in a region around some point, ***h*** (***h*** = [*h*_1_ *h*_2_ *h*_3_ *h*_4_ *h*_5_]), then we may express the fMRI matrix, **V**, as an expansion of the MEG matrices **W**_*f*_ using a multivariate Taylor series.

In general, for vector functions, a multivariate Taylor series with five dependent variables up to second order terms can be expressed as

(2a) $$F\left(x\right)=F\left(h\right)+{\left(\nabla F\left(h\right)\right)}^{T}\left(x-h\right)+\frac{1}{2}{\left(x-h\right)}^{T}H\left(h\right)\left(x-h\right)+R$$

where *R* is the remainder of the order *O*(***x*** − ***h***^3^), ***x*** = [*x*_1_ *x*_2_ *x*_3_ *x*_4_ *x*_5_], *T* denotes the transpose of a matrix and $||x-h||$ is the Euclidean norm of the vector ***x*** − ***h*** (Van Mieghem, 2010). The gradient vector is defined by

(2b) $$\nabla F\left(h\right)=\left(\frac{\partial F\left(x\right)}{\partial x_{1}}|{}_{x=h},\frac{\partial F\left(x\right)}{\partial x_{2}}|{}_{x=h},\ldots ,\frac{\partial F\left(x\right)}{\partial x_{5}}|{}_{x=h}\right)$$

and the 5 × 5 Hessian matrix *H*(*h*) is

(2c) $$H\left(h\right)=\left[\begin{array}{ccc} \frac{\partial^{2}F\left(x\right)}{\partial x_{1}^{2}}|{}_{x=h} & \cdots & \frac{\partial^{2}F\left(x\right)}{\partial x_{1}\partial x_{5}}|{}_{x=h}\\ \vdots & \ddots & \vdots \\ \frac{\partial^{2}F\left(x\right)}{\partial x_{5}\partial x_{1}}|{}_{x=h} & \cdots & \frac{\partial^{2}F\left(x\right)}{\partial x_{5}^{2}}|{}_{x=h} \end{array}\right].$$

If we rewrite Eq. (2a) in sum notation, we obtain

(2d) $$F\left(x\right)=F\left(h\right)+\sum_{m}^{5}\frac{\partial F\left(x\right)}{\partial x_{m}}|{}_{x=h}\left(x_{m}-h_{m}\right)+\frac{1}{2}\sum_{m}^{5}\sum_{n}^{5}\frac{\partial^{2}F\left(x\right)}{\partial x_{m}\partial x_{n}}|{}_{x=h}\left(x_{m}-h_{m}\right)\left(x_{n}-h_{n}\right)+R.$$

The expansion can be extended to ten dependent variables when combining two connectivity metrics (e.g. phase-based and amplitude-based), which will result in a 10 × 10 Hessian matrix. For Eq. (2a) we require that ||*** x*** − ***h*** || < *r*, where *r* is the radius of convergence, i.e. the radius of the largest disk in the complex plane in which the Taylor series converges (Brown et al., 1996). Note that in the present form we ignored terms larger than the second order because a multivariate Taylor series up to the third order would lead to complicated tensors and extraction of the expansion would lead to an explosion of cross-terms (Kollo and von Rosen, 2006). In addition, neurobiological interpretation of terms up to the second order is straightforward (see below).

In the specific case of mapping MEG connectivity matrices, ***W***_*f*_, onto the fMRI connectivity matrix, **V**, we assume that *F*(**W**) is indeed analytical around ***h***, where we replace ***x*** by **W**, which holds when the eigenvalues *λ* of the matrices ***W***_*f*_ obey ||* λ* − *h*_*f*_ || < *r* (Van Mieghem, 2010). We consider ***h*** = 0 in our estimations for two reasons: (1) this choice for ***h*** is close to the data points. (2) Since *trace*(**W**_*f*_) = 0, therefore ∑λ=0 holds and so if *λ*_max_ < *r*, convergence of the series is guaranteed (see SI for an explanation about the development point in a multivariate Taylor series). In the current functional neuroimaging setting, the partial derivatives evaluated at ***x*** = ***h*** are unknown for the connectivity matrices, but these can be estimated using a fitting procedure (see Taylor series combination of weighted adjacency matrices section). Therefore, we replace the partial derivatives by scalar coefficients. The matrix version of the expansion ((2a)) in a more tractable form then reads

(3) $$F\left(W\right)=F\left(0\right)+\sum_{m=1}^{5}a_{m}W_{m}+\frac{1}{2}\sum_{n=1}^{5}\sum_{m=1}^{5}b_{\mathrm{mn}}\;W_{m}W_{n}+R.$$

The first term in Eq. (3) provides an offset to the diagonal elements in our estimated matrix. The second term corresponds to a linear combination of MEG adjacency matrices across-frequency bands. The third term contains non-linear and cross-frequency interactions.

It is important to understand the difference between linear and non-linear terms with respect to their physical interpretation. The linear term simply allows for a weighted addition of MEG derived adjacency matrices. Each individual element of a MEG adjacency matrix corresponds to the strength of a connection (i.e. amount of phase synchronization, or strength of envelope correlation) between two brain regions in some frequency band. We therefore denote this linear term ‘*direct connectivity*’ since it corresponds simply to the combination of the strength of electrophysiological connections between two brain regions. The non-linear terms however require further explanation. Mathematically, the non-linear terms (for *m* = *n* (i.e. within a single frequency band) can (accounting for diagonal symmetry) be written:

(4) $$W_{m}W_{m}=\left[\begin{array}{c} w_{1}^{T}\\ w_{q}^{T}\\ \vdots \\ w_{P}^{T} \end{array}\right]\left[w_{1}\;w_{q}\;\cdots \;w_{P}\right]=\left[\begin{array}{cccc} w_{1}^{T}w_{1} & w_{1}^{T}w_{q} & \cdots & w_{1}^{T}w_{P}\\ w_{q}^{T}w_{1} & w_{q}^{T}w_{q} & \cdots & w_{q}^{T}w_{P}\\ \vdots & \vdots & \ddots & \vdots \\ w_{P}^{T}w_{1} & w_{P}^{T}w_{q} & \cdots & w_{P}^{T}w_{P} \end{array}\right]$$

where ***w***_*q*_ represents a *P* × 1 column vector corresponding to the *q*th column in ***W***_*m*_. This means that ***w***_*q*_ contains the connectivity estimates (phase or amplitude), for a given frequency band, between brain area *q* and all other brain regions. Eq. (4) shows that, in the case where *m* = *n*, the diagonal elements of the matrix product correspond to the un-normalised variance of a given column vector, ***w***_*q*_. The off-diagonal elements correspond to un-normalised covariance between two column vectors, meaning that these values represent the overlap, or similarity, between the connectivity profiles of two brain regions. In other words, a particular matrix element, say (1, *q*), will contain a high value if the connectivity between region 1 and the rest of the brain is similar to the connectivity between region *q* and the rest of the brain. For the non-linear terms where *m* ≠ *n* we obtain cross-terms (a product of two matrices obtained from different frequency bands). The same concept of a shared connectivity profile applies but with the difference that a matrix element in the product now corresponds to similarity in connectivity profile of two brain regions in different frequency bands. In other words, matrix element (1, *q*) will be high if the connectivity between region 1 and the rest of the brain in frequency band *A* is similar to connectivity between region *q* and the rest of the brain in frequency band *B*. Overall, the non-linear term gives information about the potential contribution of shared electrophysiological connectivity profiles (within and between frequency bands) to fMRI networks. We therefore term this non-linear contribution ‘*shared connectivity*’.

Finally, note that as a further simplification for Eq. (3), we consider an error matrix **E** instead of the remainder *R* (= remainder of higher order terms) in order to compensate for the unexplained portion of the approximation. This error matrix, *E*, contains an offset for all non-diagonal elements, where **E** = *c*(***uu***^*T*^) and ***u*** being the all-one vector and *c* a scalar coefficient (similar to the approach in Meier et al. (2016)).

## Methods

We used MEG and fMRI data obtained from two different datasets and research centres.

### Subjects: dataset 1

Dataset 1 was acquired at the Sir Peter Mansfield Magnetic Resonance Centre, University of Nottingham. Thirty-one healthy control subjects (age 27.4 ± 6.4 (mean and standard deviation), 40% female) with no history of neurological impairment were originally enrolled and scanned as part of the University of Nottingham's Multi-modal Imaging Study in Psychosis. A number of subjects were excluded due to insufficient coverage in fMRI. This resulted in a total of 15 participants (age 27.7 ± 6.5 (mean and standard deviation), 60% female) in the final analysis. The study was approved by the University of Nottingham Medical School Ethics Committee, and all subjects gave written informed consent prior to participation.

### MEG data collection and pre-processing: dataset 1

MEG data were acquired using the third order synthetic gradiometer configuration of a 275 channel CTF MEG system (MISL, Coquitlam, Canada), at a sampling rate of 600 Hz and using a 150 Hz low pass anti-aliasing filter. Magnetic fields were recorded during a task-free, eyes-open condition for 10 min in a supine position. Subjects were asked to fixate on a red cross throughout. Three coils were attached to the participant's head as fiducial markers at the nasion, left and right preauricular points. These coils were energised continuously throughout acquisition to allow localisation of the head relative to the geometry of the MEG sensor array. Before MEG acquisition, the surface of the participant's head was digitised using a 3D digitiser (Polhemus Inc., Vermont). Subsequent surface matching of the digitised head shape to an equivalent head shape extracted from an anatomical magnetic resonance (MR) image (see below for acquisition details) allowed coregistration of brain anatomy to MEG sensor geometry.

Following collection, MEG data were inspected for artefacts generated by, for example, the magnetomyogram, magnetooculogram and magnetocardiogram. Any trials deemed to contain excessive interference generated via such sources were removed. In addition, trials in which the participant was found to have moved more than 7 mm from their starting position were also removed.

An atlas-based beamforming approach was adopted to project MEG sensor level data into source-space (Hillebrand and Barnes, 2005, Hillebrand et al., 2012). The cortex was parcellated into 78 individual regions according to the automated anatomical labelling (AAL) atlas (Tzourio-Mazoyer et al., 2002). This was done by registering each subject's anatomical MR image to an MNI template and labelling all cortical voxels according to the 78 cortical ROIs (Gong et al., 2009). Subsequently, an inverse registration to anatomical subject space was performed. A beamformer (Robinson et al., 2012) was then employed to generate a single signal representative of electrophysiological activity within each of these AAL regions. To achieve this, for each region, first the centre of mass was derived. Voxels were then defined on a regular 4 mm grid covering the entire region, and the beamformer estimated timecourse of electrical activity was derived for each voxel. To generate a single timecourse representing the whole region, denoted by ${\hat{Q}}_{R}\left(t\right)$, individual voxel signals were weighted according to their distance from the centre of mass such that,

(5) Q^Rt=∑iexp−ri2400Q^it,

where *i* represents a count over all voxels within the AAL region, ${\hat{Q}}_{i}\left(t\right)$ represents the beamformer projected timecourse for voxel *i*, and *r*_*i*_ denotes the distance (measured in millimetres) to the centre of mass of the region. Note that the Gaussian weighting function ensures that the regional timecourse ${\hat{Q}}_{R}\left(t\right)$ was biased towards the centre of the region. The full width at half maximum of the weighting was ~ 17 mm.

To calculate the individual ${\hat{Q}}_{i}\left(t\right)$, a scalar beamformer was used (Robinson et al., 2012). Covariance was computed within a 1–150 Hz frequency window and a time window spanning the whole experiment (excluding those trials removed due to interference) (Brookes et al., 2008). Regularisation was applied to the data covariance matrix using the Tikhonov method with a regularisation parameter equal to 5% of the maximum eigenvalue of the unregularised covariance matrix. The forward model was based upon a dipole approximation (Sarvas, 1987) and a multiple local sphere head model (Huang et al., 1999) fitted to the MRI scalp surface as extracted from the co-registered MRI. Dipole orientation was determined using a non-linear search for optimum signal to noise ratio (SNR, here computed as the pseudo-Z value (Robinson et al., 2012)). Beamformer time-courses were sign-flipped where necessary in order to account for the arbitrary polarity introduced by the beamformer source orientation estimation.

This complete process resulted in 78 electrophysiological time-courses each representative of a separate AAL region. This approach was applied to each subject individually.

### fMRI data collection and pre-processing: dataset 1

MRI data were collected using a 7 T-MRI system (Philips Achieva) with a volume transmit and 32 channel receive head coil. The anatomical MR image (used for MEG source reconstruction as well as fMRI processing) was acquired using an MPRAGE sequence (1 mm isotropic resolution, TE = 3 ms, TR = 7 ms, flip angle = 8°). Bias fields were corrected using SPM8 and brain extraction for the MPRAGE was achieved using the Brain Extraction Tool (BET v2.1, FSL (FMRIB's Software Library, http://www.fmrib.ox.ac.uk/fsl)) (Smith et al., 2004). Resting-state fMRI data were acquired using a gradient-echo echo planar imaging sequence (TR = 2 s, TE = 25 ms, flip angle = 75°, voxel dimensions = 2 × 2 × 2 mm^3^_,_ 150 volume acquisitions). Participants were asked to keep their eyes open during the scan and to fixate on a cross presented on a back projection screen and viewed through a mirror. Data were motion corrected using SPM8 (Ashburner et al., 1999). Subject-specific masks of grey matter, white matter, and cerebrospinal fluid (CSF) were obtained via automatic segmentation of the MPRAGE data (FAST v4.1 FSL (Smith, 2002)).

**Fig. B1:**
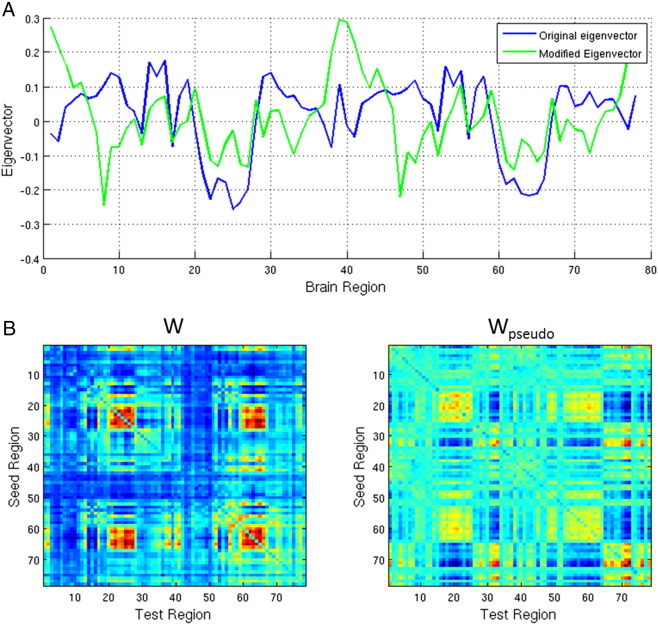
Unweighted versus weighted average over voxels in a ROI (fMRI, dataset 1). To keep our analysis consistent between fMRI and MEG, we adopted a similar pipeline for both modalities. Therefore, for fMRI, a representative time-series for a ROI was obtained by a weighted average of time-series over voxels within that ROI (as was done for the MEG analysis). Since many previous studies have used an unweighted average, we compared both approaches and found similar values for fMRI connectivity.

**Fig. B2:**
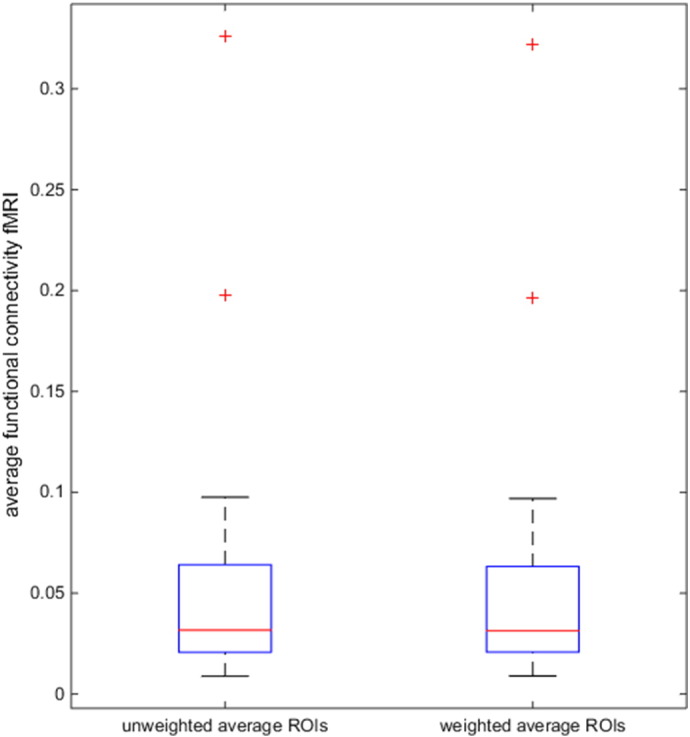
Effect of the order of correction for noise and averaging over an ROI (fMRI). We show the average fMRI functional connectivity values for both orderings i) first correction for noise followed by a weighted average over voxels in ROI, ii) first computing a weighted average over voxels followed by a correction for noise. Noise consisted of average white matter signal, average cerebrospinal fluid signal, motion during the scanning and polynomial correction. From the boxplots it can be clearly observed that the ordering did not significantly affect the results.

The AAL atlas was used to parcellate the brain into the same 78 regions of interest (ROI) as used for connectivity analysis in the MEG data (Gong et al., 2009). The fMRI data were registered to the corresponding MPRAGE image, which was in turn registered to the MNI-152 template brain (FLIRT v5.5, FSL, (Smith et al., 2004)). Inverse transformations were calculated and used to register a grey matter mask and the AAL ROIs to the functional space for each subject. These masks were then combined, to exclude white matter and CSF voxels from further analysis. In order to maintain consistency between the fMRI and MEG pipeline, a weighted average fMRI signal was computed to obtain a single signal for every ROI. This was done using the function in Eq. (5). See Fig. B1 for comparison with unweighted average over voxels in a ROI, which showed no measurable difference in connectivity between the two approaches. We then regressed out average cerebrospinal fluid signal, average white matter signal, motion and 2nd order polynomials (i.e. low frequency trends) from each regional BOLD timecourse using a general linear model in order to remove non-neuronal signals. Note that the effect of ordering (averaging and then regressing out nuisance variables or vice versa) was assessed; the results can be found in Fig. B2. The effect of average translational motion during the fMRI scan on the average functional connectivity was also assessed (Spearman correlation *R* = 0.01, *p* = 0.9).

### Dataset 2

Dataset 2 was acquired at the VU Medical Centre (VUmc), VU University, Amsterdam. Twenty-one healthy control subjects with no history of neurological impairment (age 42.5 ± 10.3 (mean and standard deviation), 65.1% female) were scanned as part of an ongoing multiple sclerosis study (Tewarie et al., 2015). The study was approved by the Ethics Review Board of the VUmc and all subjects gave written informed consent prior to participation. The data collection and pre-processing steps of the second dataset are described in a previous paper (Tewarie et al., 2015) and in the supplementary material. This dataset was used here for validation of the Taylor coefficients obtained from dataset 1. The main differences to dataset 1 with respect to MEG were 1) The instrument manufacturer (a 306 channel Elekta-NeuroMag system was employed) and 2) A peak voxel approach was employed, meaning that the voxel with maximum power in each AAL region was used as representative time-series for each ROI (as distinct from the Gaussian weighting). For fMRI, there were more pipeline differences between the two datasets: 1) A 3 T MRI system, rather than a 7 T system, was used. 2) We employed non-linear registration rather than linear registration. 3) Spatial smoothing was used, and high-pass filtering rather than polynomial regression was employed. 4) We omitted regression of average cerebrospinal fluid signal, average white matter signal and motion parameters. 5) We computed an unweighted average over voxels across each AAL region rather than a weighted average to derive a representative time signal for a ROI.

### Construction of fMRI/MEG networks

For each subject's fMRI data, we computed pairwise Pearson correlation coefficients between all possible 78 fMRI AAL signal pairs in order to obtain a symmetric 78 × 78 fMRI network, described by its weighted adjacency matrix. Negative correlation values were left intact. For MEG, we evaluated two different intrinsic modes of functional connectivity; a phase based and an amplitude based measure. Specifically, the phase lag index (PLI) (Stam et al., 2007) and the average envelope correlation (AEC) (Brookes et al., 2011a) were computed between all possible pairs of beamformer projected regional time-series to obtain symmetric 78 × 78 MEG networks for each subject. Note that this was done independently within 5 separate frequency bands (delta (1–4 Hz), theta (4–8 Hz), alpha (8–13 Hz), beta (13–30 Hz), gamma (30–48)). The PLI is a metric that captures the asymmetry of the phase difference distribution of two time-series (see SI for further details), whereas the AEC computes the correlation between the envelope of two time-series (see SI for further details; Brookes et al., 2012b, Hipp et al., 2012). Note that PLI is inherently robust to source leakage artefact. An orthogonalisation procedure (as in Brookes et al., 2012b) was employed for AEC to ensure that adjacency matrices were not dominated by the leakage artefact. Overall applying these metrics to the data resulted in 11 adjacency matrices per subject; 5 MEG based PLI matrices; 5 MEG based AEC matrices, and a single fMRI matrix. These 11 separate adjacency matrices were averaged across subjects and taken forward for further analysis. The rationale for the latter is that averaging across subjects will lead to a reduction of noise in the adjacency matrices.

### Taylor series combination of weighted adjacency matrices

**Fig. 1:**
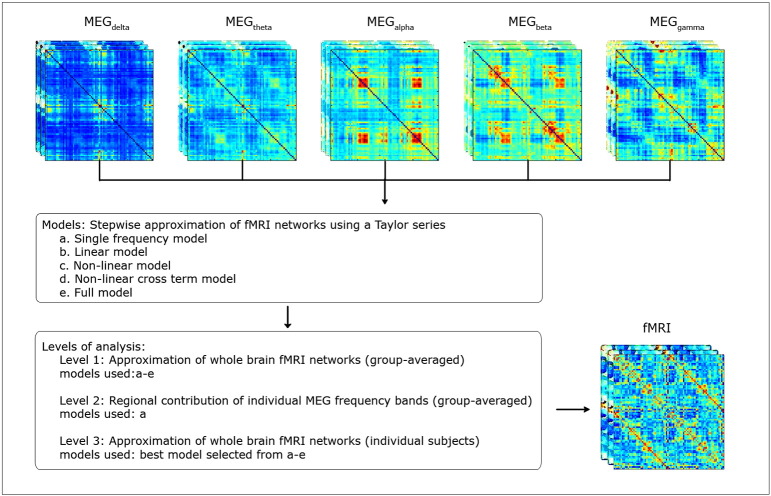
Flow chart of the analysis pipeline. For both MEG and fMRI we averaged the connectivity matrices across subjects to obtain one group averaged connectivity matrix. For MEG, this was done for each frequency band and connectivity metric separately. The MEG matrices displayed here correspond to the AEC measurement obtained from the first dataset. We then followed a step-by-step approach to approximate group averaged fMRI networks based upon MEG matrices (level 1). We included: individual frequency bands for each metric separately (model a); multiple frequency bands in a linear combination for each metric separately (model b), multiple frequency bands in a linear plus non-linear combination for each metric separately, (cross-terms excluded) (model c); Equivalent to model c but now with the cross-terms included (model d), multiple frequency bands and metrics in a linear plus non-linear combination with intact cross-terms (model e). We aimed to test the hypothesis that as models got more complex, fMRI data would be better approximated by the MEG matrices. In post-hoc analyses we examined the regional contribution of each frequency band to fMRI networks (level 2) and data fitting at the subject level (level 3).

The Taylor expansion model described in Theory section was used to study the multivariate and non-linear relationships between MEG and fMRI networks. Since the Taylor coefficients in Eq. (3) were unknown, we estimated them using an iterative non-linear least square fitting method (Byrd et al., 1987). All analyses were done using *Matlab 2013b*. Three separate levels of analysis were performed (see Fig. 1).

#### Level 1: model generation and statistical testing

We performed a sequential stepwise approximation of the fMRI weighted adjacency matrix, based upon progressively more complex combinations of the 10 MEG based weighted adjacency matrices, computed across 5 frequency bands and using 2 different connectivity metrics. Five different models were employed, based on different Taylor expansions (see also Fig. 1):

a. *Single frequency model*: fMRI matrix approximated using a single MEG matrix, belonging to a single frequency band, one FC metric (PLI or AEC), and only evaluating the linear term (direct connectivity) in Eq. (3).
b. *Linear model*: fMRI matrix approximated using MEG matrices from all frequency bands together, but using only a single FC metric (PLI or AEC) and only evaluating the linear terms (direct connectivity) in Eq. (3).
c. *Non-linear model*: fMRI matrix approximated using MEG matrices from all frequency bands together, only a single FC metric, and evaluating both the linear and non-linear terms in Eq. (3) (direct and shared connectivity), and setting the cross-frequency-terms to zero (**W**_*m*_**W**_*n*_ = 0 if *m* ≠ *n*).
d. *Non-linear cross-term model*: fMRI matrix approximated using MEG matrices from all frequency bands together, one FC metric, and evaluating the linear and non-linear terms in Eq. (3) (direct and shared connectivity), with the inclusion of cross-frequency-terms.
e. *Full model*: fMRI matrix approximated using MEG matrices from all frequency bands together, using both FC metrics, and evaluating the linear and non-linear parts in Eq. (3) (direct and shared connectivity), with the inclusion of cross-frequency-terms.

For models a-e above, the success of combined MEG matrices in predicting the fMRI matrix was evaluated using a goodness-of-fit measure (R^2^).

We aimed to test two separate hypotheses:

1) MEG derived matrices, combined using all of the Taylor based models listed above, predict a significant amount of variance in the fMRI matrix.
2) Moving to progressively more complex models (i.e. adding extra terms) significantly improves prediction of the fMRI matrix.

In order to test these two hypotheses statistically, we employed a permutation approach in which pseudo-matrices were generated. To obtain these pseudo-matrices we first performed an eigenvalue decomposition of the real MEG derived matrices. Each eigenvector was then randomised using a phase based technique (O'Neill et al., 2015, Prichard and Theiler, 1994) (see Appendix A for further details). Reconstruction of the matrix post-randomisation yielded a pseudo-matrix, similar in mathematical structure to the genuine adjacency matrices, but not reflecting genuine MEG derived functional connectivity. Using these pseudo-matrices we performed the following tests:

• *Test 1*: To test hypothesis 1 we employed 1000 iterations of a permutation test (Nichols and Holmes, 2002). On each iteration, a new set of 10 MEG pseudo matrices were generated (each based upon the 10 genuine MEG derived matrices). They were combined using the Taylor expansion for all models (a-e above), and the extent to which they could predict the fMRI matrix was measured via the R^2^ value. This generated an empirical null distribution based upon 1000 R^2^ values denoting the extent to which fMRI connectivity could be predicted by pseudo-matrices. Comparison of this null distribution with the genuine R^2^ value (from the real MEG matrices) then allowed calculation of a p-value representing the probability that variance explained in fMRI by models a-e above could have occurred by chance. Results were considered significant at a p-value of up to 0.05, corrected for multiple comparisons (Bonferroni) for the 5 separate tests over all five models.

Testing hypothesis 2 is non-trivial, since it is always the case that adding more terms to a model would likely improve variance explained. Two separate tests were run:

• *Test 2a*: We first derived R^2^ values from each of the models (a–e) using real MEG derived matrices. A gradient was measured representing the rate of improvement of R^2^ with increasing model complexity. Across 1000 iterations, we then constructed a null distribution where this same gradient was measured, but using ‘sham’ R^2^ values derived from pseudo-matrices. Note that for the ‘sham’ R^2^ values we would also expect an improvement in variance explained with increasing model complexity, and therefore a positive gradient (since adding additional terms usually leads to more variance explained). However rejection of the null hypothesis would suggest that the rate of improvement observed in real data did not occur by chance. Comparison of this empirical null distribution with the genuine gradient allowed calculation of a p-value. Results were considered significant at p < 0.05.
• *Test 2b*: For each of the 3 increments in model complexity (1: moving from a single frequency to a linear model; 2: moving from a linear to a non-linear model; 3: moving from a non-linear to a non-linear plus cross term model) we tested whether each step generated a significant increase in R^2^. To do this, we first measured the difference in R^2^ between successive models using the MEG derived matrices. Again over 1000 iterations, we then measured the same difference using pseudo-matrices, thus constructing a null distribution. Comparison of the null distribution with the genuine R^2^ difference allowed calculation of a p-value. Results were considered significant at a p- value of less than 0.05, corrected for multiple comparisons (Bonferroni) for the 3 separate tests.

These three separate tests (1a, 2a, 2b) allowed direct testing of our two primary hypotheses. Finally, in order to further validate our Taylor models, we measured correlation in the Taylor parameters (i.e. *a*_*m*_, *b*_*mn*_ from Eq. (3)) derived via application of the models to dataset 1 and dataset 2. Here, we reasoned that if the models used were genuinely reflective of an MEG to fMRI network mapping, then the parameters would be significantly correlated across these two completely independent datasets.

#### Level 2: the contribution of each frequency band

Given the prior knowledge that MEG networks show frequency specific structure, we expected to see the same patterns in the prediction of fMRI networks. Therefore, we examined the contribution of each MEG frequency band separately to the fMRI network by inspecting the regional connections explained by each band. Results were based on the approximation from our single frequency model (model a), and the percentage of connections shown was based on the obtained R^2^ (e.g. for R^2^ = 0.1, top 10% of connections displayed). These analyses were only done for model *a* since the end results of estimations from the other models were obtained by a weighted sum of all frequencies.

#### Level 3: individual subject analysis

The most accurate model from level 1a–d was used within each individual subject in order to address how well MEG weighted adjacency matrices computed within a single individual can predict their fMRI counterpart. PLI and AEC obtained results were compared using a Mann–Whitney U test. In order to assess this statistically, we performed permutation analysis (Nichols and Holmes, 2002). We reasoned that if the two modalities contained subject specific information, then the MEG derived networks from subject *A* would be a better predictor of the fMRI network from subject *A*, compared to, for example, the MEG networks from subject *B*. To this end, we swapped MEG networks randomly across subjects to get unmatched pairs of MEG and fMRI networks, from which a null-distribution of R^2^ values was generated (N = 1000 permutations). The genuine R^2^ was compared against this null distribution using a significance level of *5*%.

## Results

### Approximation of group level fMRI networks by MEG networks

**Fig. 2:**
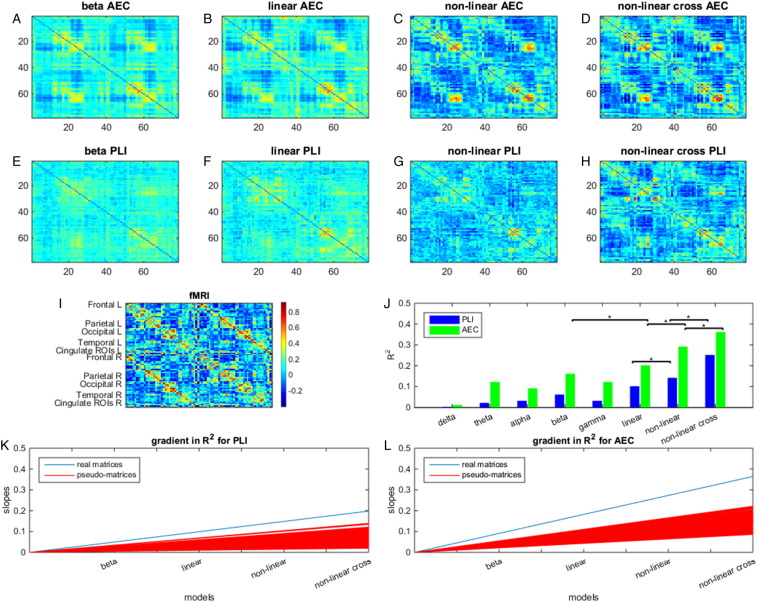
Stepwise approximation of fMRI networks through Taylor series: Dataset 1. fMRI network approximations are displayed from the left to the right. The first row shows approximations based on the AEC (A–D), the second row shows approximations based on the PLI (E–H) and the fMRI network is displayed in the third row (I) together with a colour bar that is the same for all the matrices shown. The ROIs are ordered according to Gong et al. (2009). Results are shown, from left to right, for the single frequency model (A, E), the linear model (B, F), the non-linear model (C, G), and the non-linear cross-term model (D, H). In the third row, the bar chart shows the R^2^ values using either combinations of AEC matrices (green) or PLI matrices (blue) (J). A clear improvement in explained variance can be seen as more terms are included in the model, i.e. when moving from the specific frequency band predictions, to the approximations that include multiple frequency bands, nonlinearity and cross-terms. This is the case for both the AEC and PLI. These improvements are significant beyond chance, as can be seen by the results of the permutation tests; here * denotes statistical significance (p < 0.015; Bonferroni corrected for three tests; test 2b). Note that only the best single frequency model (beta band) was included for the analysis). This stepwise improvement is also apparent from the estimated fMRI matrices (first and second row), with the best approximations in the right hand column (E, H). This can be seen from the increasing number of strong connections near the diagonal and the two off-diagonals. A gradient for the genuine rate of improvement of R^2^ (blue) with increasing model complexity and rate of improvement from 1000 permutations (red) is depicted in (K) for PLI (test 2a; p < 0.001) and in (L) for AEC (test 2a; p < 0.001).

**Fig. 3:**
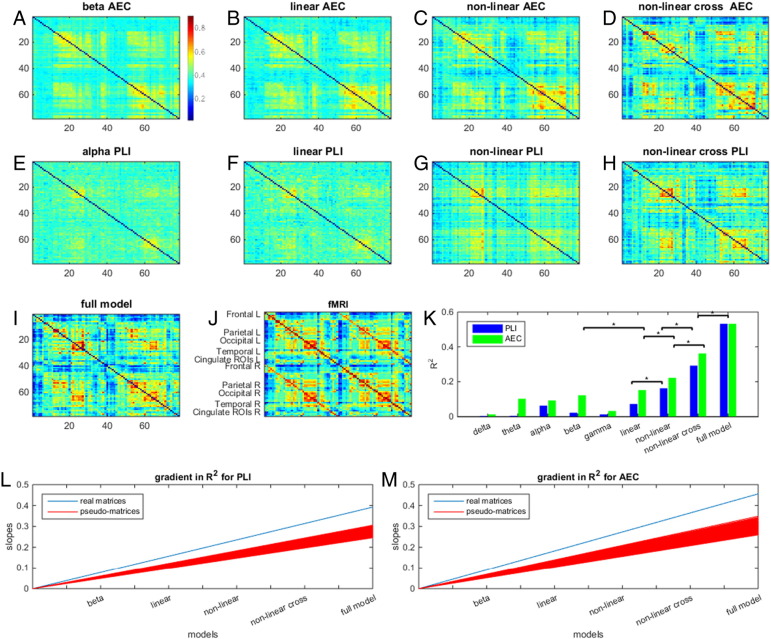
Stepwise approximation of fMRI networks through Taylor series: dataset 2. Note that this figure is equivalent to Fig. 2, but is constructed using dataset 2. The figure shows fMRI network approximations based on AEC (A–D), and PLI (E–H). Results are shown, from left to right, for the single frequency model (A, E), the linear model (B, F), the non-linear model (C, G), the non-linear cross-term model (D, H) and the full model (I), together with a colour bar that is equivalent for all matrices. The fMRI matrix is displayed in (J). In the third row, the bar charts show R^2^ values using either combinations of AEC matrices or PLI matrices (K). As with dataset 1 (Fig. 2), a clear improvement in explained variance can be seen as more terms are included in the model. Again * denotes significant (p < 0.015; Bonferroni corrected for three tests) improvement in R^2^. A gradient for the genuine rate of improvement of R^2^ (blue) with increasing model complexity and rate of improvement from 1000 permutations (red) is depicted in (L) for PLI (test 2a; p < 0.001) and in (M) for AEC (test 2a; p < 0.001).

Using our expansion framework, our primary aims were to test firstly whether MEG derived matrices, combined using all of the Taylor based models listed in Fig. 1, predict a significant amount of variance in the fMRI matrix. Secondly we aimed to test whether moving to progressively more complex models (i.e. from single frequency to multiple frequencies; linear to non-linear, within band to within and between band, and from single connectivity metric to using two connectivity metrics) significantly improves prediction of fMRI based functional connectivity. These primary results are shown in Fig. 2, Fig. 3:

Overall, our results confirm our two hypotheses. Firstly, all of the models used, even the single frequency model, were able to predict significant variance in the fMRI connectivity matrix when compared to the empirical null distributions (test 1 null hypothesis rejected). This simple finding adds weight to previous papers showing a significant overlap between fMRI and MEG based connectivity matrices, even when only single frequency bands are used (Liljeström et al., 2015, Tewarie et al., 2014). Secondly, adding 1) multiple frequency bands together, 2) non-linear interactions (shared connectivity) and 3) cross-frequency terms led to significantly better prediction of the fMRI matrix; this was shown by the measured gradient, depicting the increase in R^2^ across progressively more complex models, being significantly larger for real data compared to the pseudo-networks (test 2a null hypothesis rejected). Secondly, when measuring the improvement in R^2^ for each incremental increase of model complexity, we observed a significant increase in explained variance. This was the case for all three model increments for AEC, and 2 out of the three for PLI (test 2b null hypothesis rejected). It proves helpful to now discuss each model in detail.

**Fig. B3:**
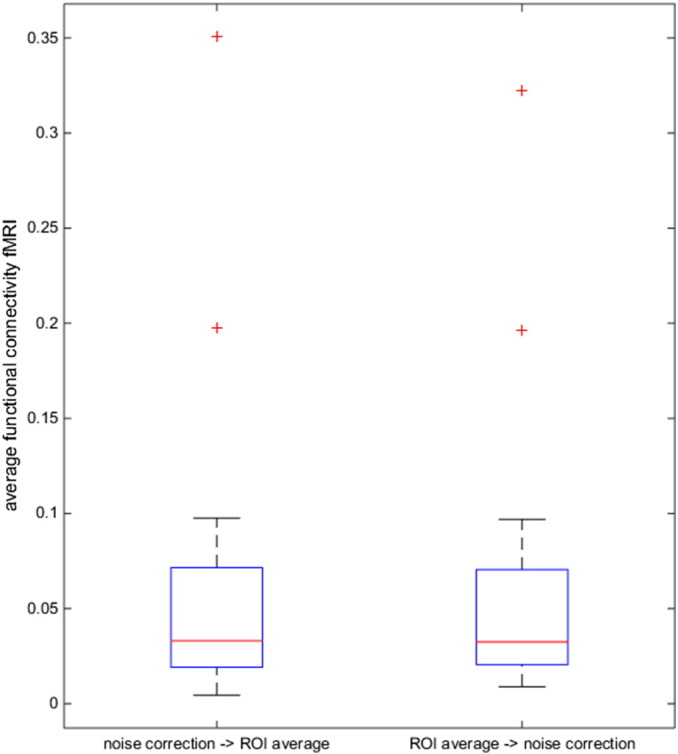
Estimated parameters (Taylor coefficients) for analysis level 1 using model b, linear approximation based on all MEG frequency bands using the AEC as functional connectivity metric for MEG. Note that the confidence intervals of the estimated Taylor coefficients for the two datasets almost completely overlap.

**Fig. B4:**
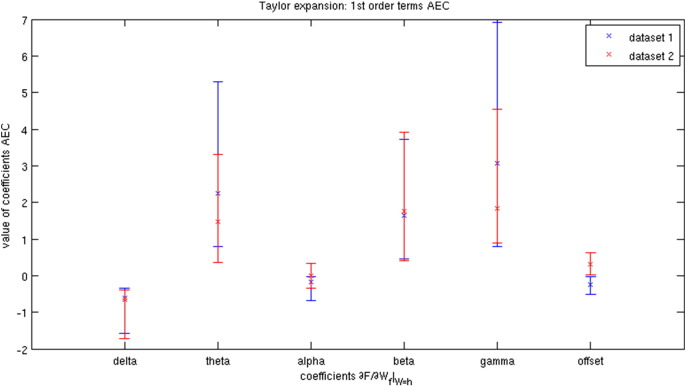
Estimated parameters (Taylor coefficients) for analysis level 1 using model b, linear approximation based on all MEG frequency bands using the PLI as functional connectivity metric for MEG. Note that the confidence intervals of the estimated Taylor coefficients for the two datasets almost completely overlap.

**Fig. B5:**
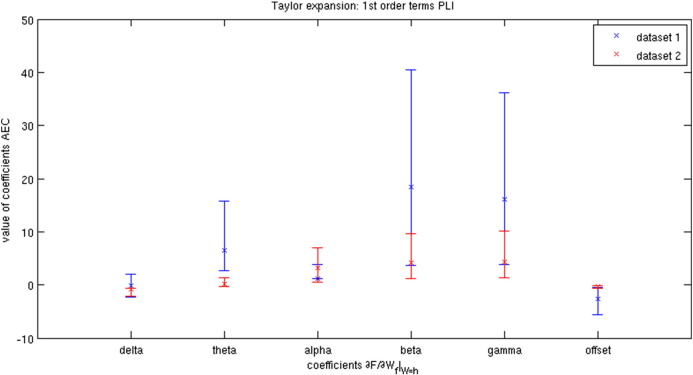
Estimated parameters (Taylor coefficients) for analysis level 1 using model d, linear + non-linear approximation based on all MEG frequency bands using the AEC as functional connectivity metric for MEG. Note that the confidence intervals of the estimated Taylor coefficients for the two datasets largely overlap.

**Fig. B6:**
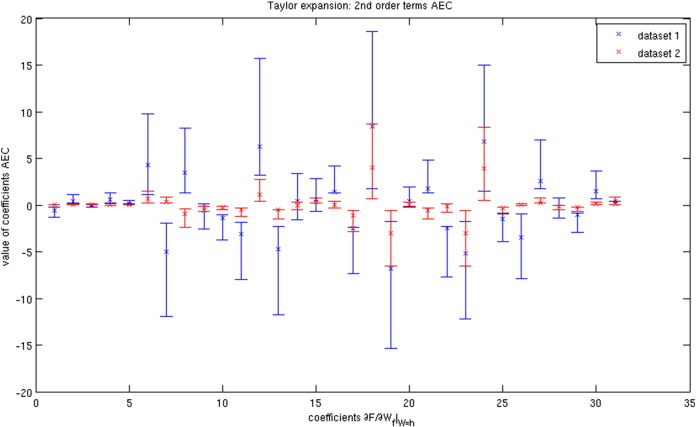
Estimated parameters (Taylor coefficients) for analysis level 1 using model d, linear + non-linear approximation based on all MEG frequency bands using the PLI as functional connectivity metric for MEG. Note that the confidence intervals of the estimated Taylor coefficients for the two datasets sometimes overlap, but also show discrepancy between the two datasets.

**Fig. B7:**
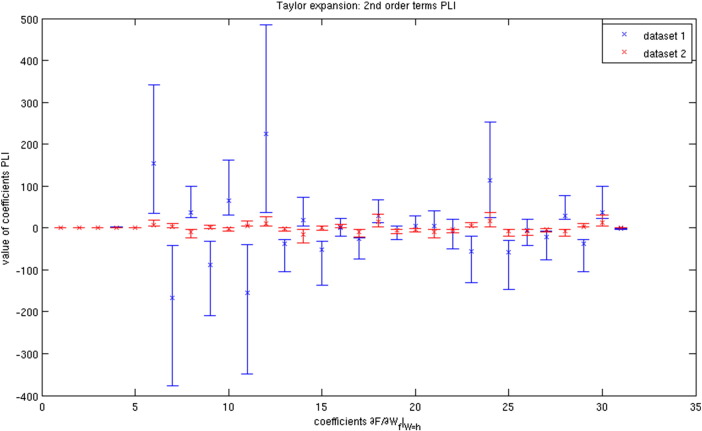
Predicted resting state network connectivity. Predicted connectivity was grouped within a RSN, and predicted connectivity between the same RSN and all surrounding regions was grouped. We display the difference in median between these two groups for the salience, sensorimotor, default mode, executive, visual and combined left and right fronto-parietal networks. The upper panel shows the AEC based prediction and the lower panel the PLI based prediction. The colours correspond to the model used, ranging from single frequency to non-linear model with cross-terms. Note that for both functional connectivity metrics, the contribution of non-linearity leads to better identification of RSNs (apart from DMN PLI). * refers to significant differences only if the difference had the right sign (within > between; p < 0.0005 (Bonferroni corrected)), x refers to significant differences with the right sign (within > between; p < 0.0005 (Bonferroni corrected)) and when the outliers were discarded in the distributions.

**Fig. B8:**
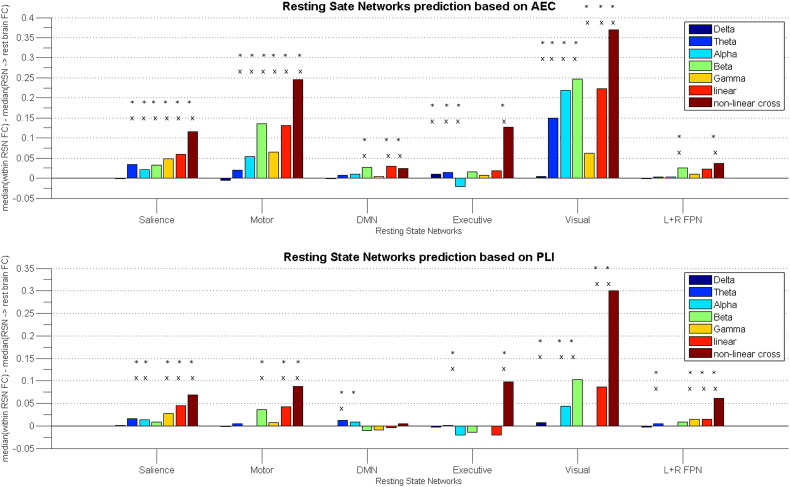
Estimated parameters (Taylor coefficients) for analysis level 1 using model e, linear + non-linear approximation based on all MEG frequency bands using the PLI and AEC as functional connectivity metrics for MEG. Note the smaller confidence intervals compared to Fig. B6, but also note that confidence intervals increase for increasing higher order terms.

‪ *Single frequency model*: When using only individual frequency bands, the beta band network, for both PLI and AEC, outperformed all other frequency bands as predictors of fMRI (first dataset; Fig. 2J). This was followed by gamma, theta and alpha network matrices for AEC and by gamma and alpha network matrices for PLI (Fig. 2J). Note that on average, the variance explained for AEC (0.01 < R^2^_AEC_ < 0.12) was higher than for PLI (0.001 < R^2^_PLI_ < 0.06). However, these low R^2^ values for PLI still explain a significantly more information in fMRI compared to pseudo-matrices. These findings were replicated in dataset 2 (Fig. 3).
‪ *Linear model*: Adding all frequency bands to the Taylor expansion, in a linear combination, led to a better approximation of the fMRI network (higher R^2^) for both AEC and PLI compared to only the beta band (R^2^_PLI_ = 0.10, R^2^_AEC_ = 0.20; Fig. 2J); however this measured improvement was only significant for AEC. Approximations made using dataset 2 showed equivalent results (R^2^_PLI_ = 0.07, R^2^_AEC_ = 0.15; Fig. 3). Note that connectivity matrices estimated by the linear model show, on average, higher connectivity values for the second dataset compared to the first dataset; this could be explained by the higher average connectivity in the fMRI matrix and/or the shorter window length used for the second dataset, which can bias the AEC/PLI towards higher values. The confidence intervals of the estimated Taylor coefficients for datasets 1 and 2 overlapped (Fig. B3, Fig. B4) and the coefficients themselves showed significant correlation for both functional connectivity metrics (*r*_PLI_(6) = 0.86 *p* = 0.03; *r*_AEC_(6) = 0.93 *p* = 0.006), indicating robustness of the mapping across two independent datasets.
‪ *Non-linear model*: We evaluated the Taylor coefficients corresponding to both linear and non-linear terms in order to investigate potential contribution of shared electrophysiological connectivity to fMRI. To exclude cross-frequency interactions, we removed the cross-terms in Eq. (3) (i.e. all *m* ≠ *n*). A significant increase in explained variance was observed for both AEC and PLI compared to including linear terms only (R^2^_PLI_ = 0.14, R^2^_AEC_ = 0.29; Fig. 2C, G). The second dataset showed equivalent results (R^2^_PLI_ = 0.16, R^2^_AEC_ = 0.22). There was significant correlation between the Taylor coefficients for datasets 1 and 2 for AEC (*r*_AEC_(11) = 0.76 *p* = 0.007) indicating robustness of the mapping. However this was not the case for PLI, *r*_PLI_(11) = 0.49 and *p* = 0.14).
‪ *Non-linear model with cross-terms*: We repeated the non-linear model, but with cross-terms retained, which allows examination of the contribution of cross-frequency shared connectivity to fMRI. Adding cross-frequency terms led to significantly better fMRI network approximations for both dataset 1 (R^2^_PLI_ = 0.25, R^2^_AEC_ = 0.36; Fig. 2J) and dataset 2 (R^2^_PLI_ = 0.29, R^2^_AEC_ = 0.36; Fig. 3K), for both PLI and AEC, compared to the approximation when cross-terms were ignored. The Taylor coefficients and their confidence intervals for the approximation based on the AEC again largely overlapped and correlated significantly (*r*_AEC_(30) = 0.48, *p* = 0.007; Fig. B5). For PLI this was not the case (*r*_PLI_(30) = 0.26, *p* = 0.15; Fig. B6) since the Taylor coefficients of the first dataset displayed large confidence intervals. For all analysis levels up to the linear cross-term model we analysed whether we could observe standard resting state networks (RSNs) in the whole brain approximations as credibility check for our results (e.g. default mode-, sensorimotor-, salience-, fronto-parietal-, executive-, and the visual-network). The clearest RSN patterns could be observed for AEC in the approximations based on the non-linear cross-term model (see Fig. B7).
‪ *Full model*: Finally, we assessed whether adding the two modes of connectivity (AEC and PLI) would improve our approximation. Since estimated parameters for PLI were associated with large confidence intervals in the non-linear cross-term model for dataset 1, we restricted this analysis to dataset 2. Note that adding PLI and AEC network matrices together into Eq. (3) resulted in ten different matrices, and therefore 110 Taylor coefficients to estimate. Given the number of matrix elements $\left(\frac{N^{2}-N}{2}=3003\right)$, the number of coefficients is still relatively small so that overfitting based on numerous parameters is not an issue. After evaluation of Eq. (3) we obtained an R^2^ = 0.53 (Fig. 3I and K). Although the current fit involved estimation of 110 coefficients, the confidence intervals were still small and did not become unstable, as was the case for the analysis with PLI (non-linear cross-term model) for dataset 1 (Fig. B8).

**Fig. 4:**
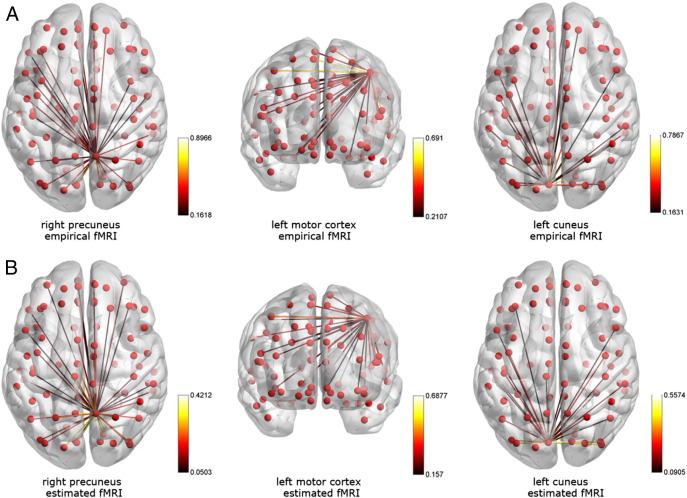
Estimated fMRI connections for individual regions. Arbitrary thresholded fMRI connections for three regions (right precuneus, left motor cortex and left cuneus) are shown in (A) on a template mesh. Panel (B) shows connectivity from the same seed regions based on the non-linear cross-term model, obtained with AEC.

Overall, results show that, using AEC, the best model for fMRI connectivity results from the non-linear model with cross-frequency terms included. This predicted significantly greater variance in the fMRI network matrix than the other models, and extracted Taylor series parameters correlated significantly across the two independent datasets. These results are visualised in Fig. 4, which shows a simple comparison (using AEC in dataset 1) of connectivity patterns, derived from fMRI and the combined MEG model, for three arbitrarily chosen but strongly connected AAL regions (right precuneus, left motor cortex and left cuneus). Note that the connectivity profiles measured in fMRI, and using the non-linear cross term model applied to MEG, were similar for all three chosen brain regions.

In contrast, PLI was somewhat more variable. This was particularly the case for the non-linear cross term model in dataset 1, where parameter estimation was unstable. However, the generic finding showed a significantly better fit to fMRI with increasing model complexity, implying again that shared connectivity contributes significantly to fMRI connectivity.

### Regional contribution of individual MEG frequency bands

**Fig. B9:**
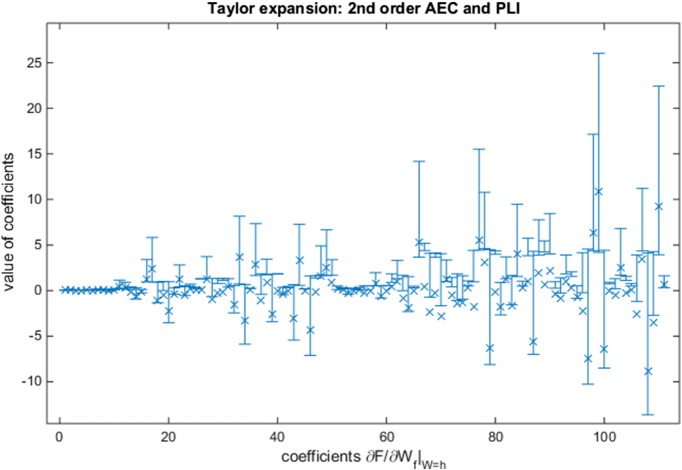
Regional contribution of individual MEG frequency bands (PLI; first dataset). The linear approximations based on individual frequency bands (model a) are shown in more detail. The upper panel illustrates the predicted connections, where the threshold for the number of connections is based on the R^2^. The bottom panel shows the predicted average connectivity per ROI, i.e. node strength/degree. Note that the predicted networks are sparser than the AEC predictions (Fig. 5). Nevertheless, the beta and alpha show more occipital and temporal connection patterns, whereas theta and gamma show more frontal and parietal connections. The PLI gamma band shows a distinct pattern compared to its AEC counterpart, where a fronto-temporal pattern of connections could be observed.

**Fig. 5:**
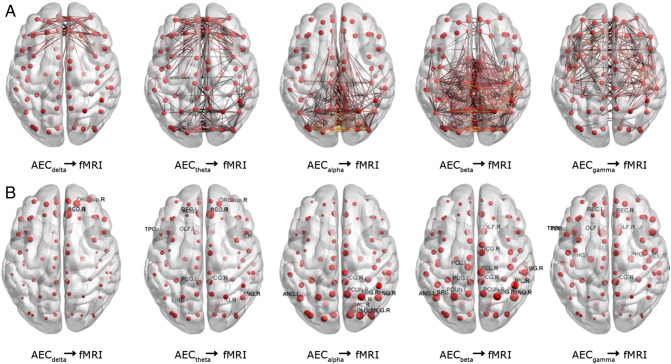
Regional contribution of individual MEG frequency bands. The linear approximations based on individual frequency bands (single frequency model) are shown in order to understand the regional contribution of each MEG frequency band to fMRI. Results are shown for estimations using AEC (dataset 1). The upper panel illustrates the predicted connections, where the threshold for the number of connections shown is based on R^2^ (e.g. for R^2^ = 0.1, top 10% of connections displayed). The size of the spheres in the bottom panel denotes the predicted average connectivity per ROI, i.e. node strength. Note that, in agreement with the results for the whole network analysis (Fig. 2, Fig. 4) the beta band connections were able to predict most fMRI connections, with dominant patterns in parietal, sensorimotor, occipital and temporal areas. Note the more frontally dominated patterns for the delta and gamma band, and split pattern in the theta band.

Using the single frequency model, we examined to what extent MEG networks in individual frequency bands were able to predict fMRI. Fig. 5 shows the predictions for all frequency bands using AEC (dataset 1). The fraction of connections shown in each graph is based on the R^2^ value calculated (e.g. for R^2^ = 0.1, threshold of 10% connections; Fig. 5A). Fig. 5B shows the average functional connectivity for each ROI for the different frequency bands. Results reveal that different MEG frequency bands explain specific regional connections. The delta band predominantly explains fMRI connections in frontal areas. Theta AEC networks show dominant patterns in frontal and occipital areas whilst connections explained by alpha band AEC are predominantly posterior. Beta band AEC was able to explain 16% of the variance in the fMRI matrix. Especially parietal, sensorimotor and occipital as well as temporal fMRI connections were explained by the beta band. Lastly, the gamma band AEC explained fMRI structure in frontal areas. For PLI, the R^2^ values were generally lower and therefore fewer connections were displayed (see Fig. B9).

### Approximation of fMRI networks at the subject level

**Fig. 6:**
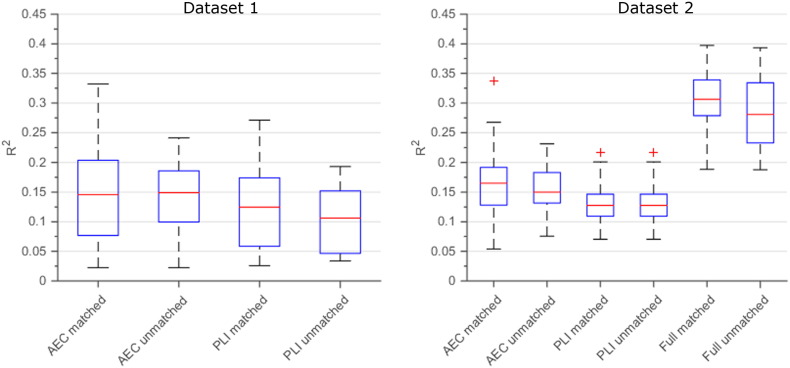
Subject level prediction of fMRI networks. Illustrated are predictions using our non-linear cross-term model for individual subjects, for both PLI and AEC. For dataset 2, results from the full-model are also illustrated. For both datasets the R^2^ values are shown for the matched subject pairs as well as one realisation for the unmatched (permuted) pairs. Note that the R^2^ values of the matched pairs do not outperform the unmatched pairs.

Finally, we applied the same Taylor series expansion approach to estimate fMRI networks for each individual subject. For both datasets and both FC metrics we evaluated Eq. (3) using the non-linear model with cross-terms. For dataset 2, individual predictions based on the AEC performed better than predictions based on the PLI (Mann–Whitney *Z* = 1.98, *p* = 0.05; Fig. 6). However, this was not the case for dataset 1 where the distributions of the explained variances were similar (Mann–Whitney *Z* = 0.93, *p* = 0.35). Note that for both datasets the individual predictions were generally lower than the group-level predictions (compare R^2^ values of Fig. 6 with R^2^ values of Fig. 2J and Fig. 3K). For dataset 2, we also evaluated Eq. (3) using the full model. Individual predictions based on this model were significantly better than predictions based on the non-linear model with cross-terms (Mann–Whitney *Z* = 5.13, *p* < 0.001). We performed a permutation analysis by swapping individual fMRI and MEG networks to investigate if the fMRI network of a specific subject was better predicted by his or her MEG data, compared to MEG data from another individual. However, no subjects showed a significant result (Fig. 6), for either the non-linear model with cross terms or the full model.

## Discussion

In this study we investigated the relationship between electrophysiological and haemodynamic networks, using a unique mathematical framework based upon the assumption that the relationship between MEG and fMRI can be considered as a mathematical function that can be analysed using a multivariate Taylor series. This framework allowed us to integrate MEG data from multiple frequency bands and connectivity metrics, together with linear and non-linear interaction terms, to predict fMRI networks. Our main finding is that, although single frequency band MEG derived networks explain significant variance in the fMRI network matrix, the accuracy of predicted fMRI networks drastically improved when we considered the multivariate, linear, non-linear and cross-frequency combinations of MEG features.

Using a single frequency model, we were able to find spectral specificity in regional fMRI connections. Overall, the beta frequency band was the best predictor of fMRI connections in both AEC and PLI and this finding is in agreement with earlier studies that have generally shown significant agreement between fMRI and MEG beta band derived resting state networks (see Hall et al., 2014 for a review). Here (Fig. 5) we have shown that parietal, sensorimotor, occipital and temporal fMRI connections were well explained by MEG beta band networks. Interestingly however, frontal connections in fMRI were better explained by the theta and gamma frequency bands, whereas the alpha band predominantly explained occipital/parietal connectivity. Anterior–posterior connections were observed in both the alpha and theta bands, which has also been shown in a previous directed connectivity study (Hillebrand et al., 2016). The presence of frequency specific regional connections, and their regional distribution, is in line with recent work on the relationship between MEG and fMRI (Hipp and Siegel, 2015). Overall this implies that fMRI must be seen as an integral of multiple electrophysiological networks that occur on a variety of temporal scales.

The spatial inhomogeneity in MEG connectivity across-frequency bands suggests that integrating multiple frequencies into a single description, using a linear model, would improve prediction of fMRI. We used our Taylor model to show that, for envelope based networks, this was indeed the case with a significant improvement in R^2^ when using a linear combination of frequency bands. Therefore, fMRI networks may well result from a combination of frequency bands, where each separate band adds regionally specific information (Mantini et al., 2007). In regions where two frequency bands show similarity, for example in occipital areas where alpha and beta connections overlap, an fMRI connection could be considered as a weighted sum across those bands. Whilst PLI showed improvement in R^2^ between the single frequency and the linear model, this failed to reach significance. It is important to note that the estimated Taylor coefficients for the linear model suggested that all frequency bands were represented, with no disproportionally high coefficients, and no bands that could be neglected. This said, delta band connectivity consistently contributed the least to fMRI. Importantly, the linear contribution of separate frequency bands to the fMRI predictions was consistent between both datasets; this was shown by both overlap in estimated Taylor coefficients as well as a significant correlation between them. This result is extremely important as it underlines the robustness of our mapping approach.

Adding non-linearity to the Taylor expansion significantly improved our approximation of fMRI networks; this was true for both AEC and PLI. Importantly, this was not simply the result of adding increased model complexity, since this was accounted for in our statistical testing. The non-linear models included quadratic terms for each individual frequency band as well as cross-terms between frequency bands. Here the independent addition of both generated a significant improvement in fMRI prediction. The non-linear terms effectively measure covariance between the neuronal connectivity profiles of separate regions (see Eq. (4)). The physical interpretation of the non-linear terms helps explain why their addition improves the prediction of fMRI matrices: If two regions are electrophysiologically interacting with similar areas, i.e. share the same connectivity profile, then it is likely that their energy demands (i.e. their BOLD signals) are influenced in a similar fashion by those shared connections. This likely increases the temporal correlation between BOLD signals, and hence increases haemodynamic functional connectivity. The effect of shared connectivity profiles on BOLD correlations also extends to cross-frequency terms. The reader should note that the cross-frequency terms in our model do not contain direct cross-frequency coupling between regions (e.g. a direct link between, for example, alpha in region 1 and gamma in region 2, as is often derived in, e.g. phase-amplitude coupling (Aru et al., 2015, Jensen and Colgin, 2007)). Rather, our cross-frequency terms correspond to shared connectivity patterns between independent networks existing within different frequency bands. Their interpretation is therefore similar to that for non-linear terms within frequency bands. Overall, our result suggests that BOLD connectivity results from not only direct neuronal connectivity (i.e. an electrophysiological connection between the two regions in question) but also shared connectivity profiles both within and between frequency bands. This important result should be considered in future multi-modal connectivity studies.

**Fig. B10:**
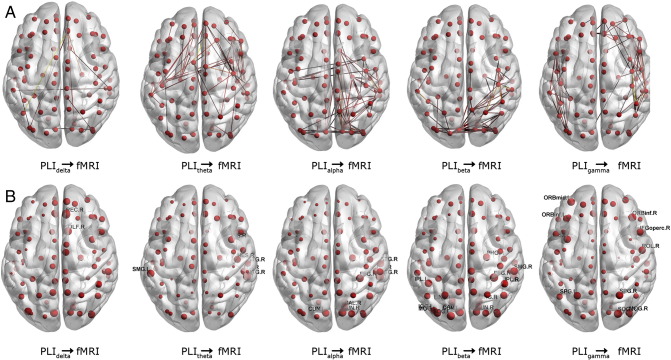
MEG connectivity matrices for the first dataset. Illustrated are the AEC (upper row) and PLI (bottom row) connectivity matrices for the different frequency bands. Note that the AEC matrices contain clearer structure. Structure in the lower frequency bands is less clear, and different for the lower frequency bands for PLI compared to AEC. The alpha and beta band PLI and AEC matrices contain similar patterns, i.e. four “blocks” around areas 20 and 60, which are posterior regions. For the gamma band, some patterns are similar between AEC and PLI (blocks around ROIs 30 and 50), but there are also notable AEC connections that are absent in the PLI matrix, and vice versa. Notice that the PLI matrix is transformed by taking the natural logarithm of all values to increase the range of the values.

**Fig. B11:**
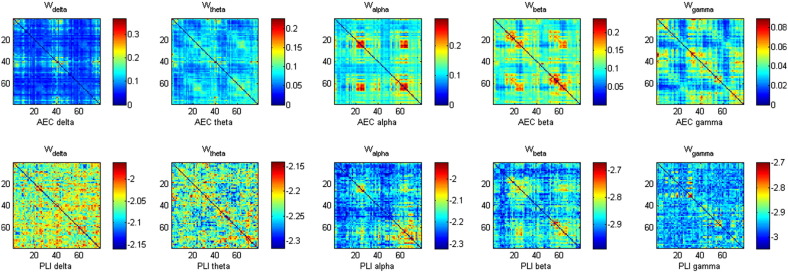
MEG connectivity matrices for the second dataset. Illustrated are the AEC (upper row) and PLI (bottom row) connectivity matrices for the different frequency bands. Note that the AEC matrices contain clearer structure. The alpha and beta band PLI and AEC matrices do seem to contain similar patterns, i.e. four blocks around areas 20 and 60 which are posterior regions. The gamma band lacks structure for both functional connectivity matrices. Note that in comparison to Fig. B10 the gamma band connections are less clear, in contrast to theta band connections which are more prominent. This could have several reasons, such as MEG system type, eyes-open versus eyes-closed recordings or differences in cohorts. Notice that the PLI matrix is transformed by taking the natural logarithm of all values to increase the range of the values.

In our Taylor series approximations, we also evaluated the role of the different intrinsic coupling modes (phase (PLI) and amplitude (AEC)). Separate analysis for the AEC and PLI revealed that AEC derived networks were better predictors for fMRI than PLI derived networks. This was the case for all models (single frequency, linear and non-linear (with and without cross-terms), and for both datasets). This result is also in agreement with previous work on phase versus amplitude based interhemispheric sensorimotor network coupling (Brookes et al., 2011a). The reason for this might be twofold: Firstly, the AEC networks show more spatial structure than the PLI networks, which have a more random appearance (see Fig. B10, Fig. B11 for spatial structure of the MEG matrices). This higher noise would likely lead to less explained variance for the PLI estimations. The more random appearance of PLI could result from multiple effects. This could be related to the fact that larger phase differences are needed for AEC than for PLI in order to be able to detect a functional connection (Hipp et al., 2012), and therefore the AEC matrices might contain more false negative values and PLI more false-positive values, leading to a more structured network for AEC. The more random appearance of PLI compared to AEC is certainly a consistent feature of all data in this study, and future investigation using 1) voxel rather than regional time-courses and 2) non-stationary rather than stationary connectivity might shed light on this observation. A second possible reason for the close relationship between AEC and fMRI may be that AEC is based on the envelopes of a time-series, which evolves on a slower time-scale than the phase information; it may therefore be more closely related to the BOLD signal. This said, combining AEC and PLI does add information in terms of explaining fMRI networks: Using our full model, we combined AEC and PLI in a multivariate non-linear approximation and this led to a higher explained variance than an approximation based on AEC alone (or PLI alone), indicating that fMRI networks also reflect the sum of amplitude and phase interactions. However, note that a multivariate non-linear approximation with two connectivity measures only gave a maximum R^2^ of 0.5. It is possible that addition of higher order terms would improve the approximation, or that the unexplained variance in fMRI is the result of non-neuronal signal (Birn et al., 2008), noise in the MEG and fMRI measurements, or differences in the spatially inhomogeneous signal-to-noise ratio of both modalities.

Finally, our analysis included approximation of fMRI networks at the individual subject level. Using our non-linear model with cross terms included, we were able to predict variance in fMRI connectivity matrices within individual subjects, although these approximations were not as good (in terms of variance explained) as their group level equivalent. In addition, results suggested that a subject's own MEG networks were no better at predicting their fMRI than MEG networks derived from different subjects. The reason for this is unclear: it could be that, since connectivity matrices are so well matched across individuals, any inter-individual differences are lost in noise. In fact, a previous study supports this lack of subject specificity between fMRI and MEG networks at the global level (Hipp and Siegel, 2015). However, it is also important to note that relatively poor within subject reliability of MEG connectivity measurements has been shown previously. For example, Wens et al. (2014) show that whilst group level static connectivity within several well-known distributed networks is stable, there is significant variability at the individual subject level. Such variability may originate from a number of sources including artefacts in the MEG data, source modelling and connectivity estimators. Given such findings of large inter-individual differences, it is not necessarily surprising that our individual measures do not offer extra insight in predicting fMRI measurements. This said, ultimately, if techniques like the one presented here are to be useful clinically, then we must derive means to ensure their robustness in individuals. Further effort is thus needed in this area.

### Methodological considerations

The key assumption underlying our model is that the relationship between MEG and fMRI can be described by an analytical multivariate mathematical function. Although we did not verify that our function is indeed analytical, there is good reason to expect that our assumption is valid. Firstly, our fitted Taylor coefficients were highly stable across multiple iterations of the fitting algorithm. Secondly, our fitting using real MEG derived adjacency matrices consistently outperformed equivalent fitting using the pseudo-matrices; this also showed that our obtained increase in goodness-of-fit values was not simply the result of increased model complexity. Finally, when deriving our Taylor coefficients using two completely independent multi-subject datasets, we observed significant correlation between the fitted Taylor coefficients, showing definite structure to the estimated parameters that relate directly to the function itself. This critical final point shows the robustness of our fitting; given the significant differences between the two datasets in terms of both acquisition and analysis, it is very comforting that entirely different processing pipelines yield significantly correlated mapping parameters which are not affected by scanner type or processing pipeline. It should of course be noted that neither correlation nor overlap of estimated parameters were perfect. Also the adjacency matrices between the datasets differed. Indeed, both the imperfection of the overlap and the difference in matrices could be due to differences in analysis pipelines, data acquisition, MRI scanner type/magnetic field strength, MEG system type, eyes-closed versus eyes-open condition during MEG acquisition and even gender and age differences between the cohorts used for the datasets. This difference between datasets also hampered our use of cross-validation analysis, which is a procedure whereby the estimated parameters from one dataset are applied on another dataset to check for generalisation of a model. Here, it appeared that there was more common mode interference in dataset 2, leading to generally higher connectivity estimates in fMRI (likely a result of lower magnetic field strength and different analysis pipeline). This means that the range of the correlation coefficients was reduced in dataset 2, with genuine physiological variation across the brain occupying a smaller range in dataset 2 compared to dataset 1. This indicates that a straightforward swap of Taylor coefficients between datasets is not applicable, but correlation between Taylor coefficients is, since the correlation is not affected by the magnitude of parameters, only the pattern.

## Conclusion

In conclusion, we have, for the first time, employed a multivariate Taylor expansion framework to investigate the relationship between networks of functional connectivity measured in MEG and fMRI. Our results show that the relationship between these two modalities extends far beyond simple mapping of frequency specific MEG networks to fMRI. In fact, fMRI connections are a reflection of direct neuronal connectivity, summed across multiple frequency bands, superimposed upon shared neuronal connectivity profiles within and between frequency bands as well as the summation of multiple modes of connectivity. Further exploration of non-linear and cross-frequency interactions will therefore shed new light on distributed networks in the task positive and resting states, and their perturbation in multiple pathologies.

## Figures and Tables

**Fig. A1:**
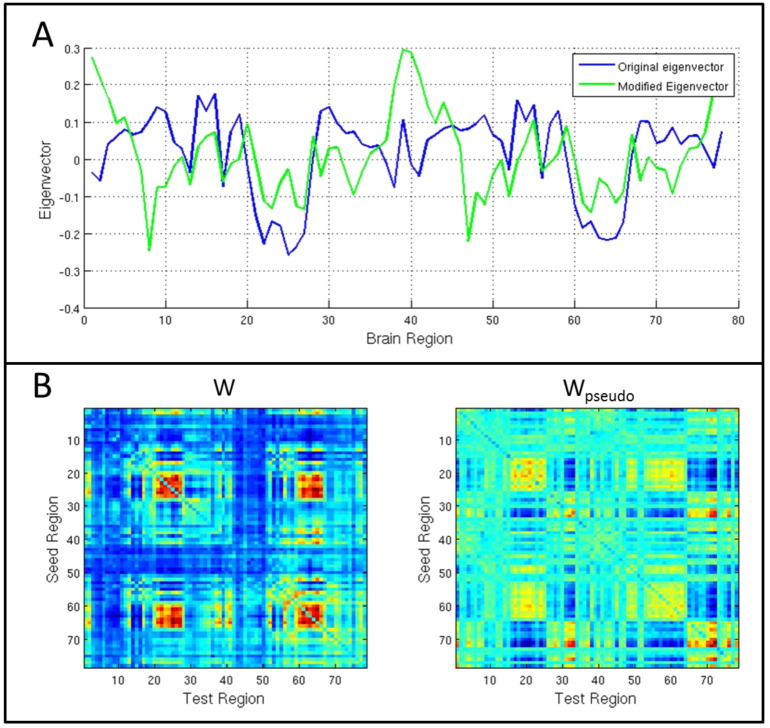
Example construction of a pseudo-matrix. A) The blue trace shows the second eigenvector of a genuine MEG alpha band AEC adjacency matrix. The green trace shows the phase randomised version. Note that the periodicity (which reflects spatial symmetry in the ordering of brain regions across the two hemispheres in the AAL parcellation) has been maintained. B) The left hand panel shows the genuine MEG adjacency matrix. The right hand panel shows a single example of a phase randomised pseudo-matrix. Note overall visual similarity in structure and smoothness.

## Appendix A Construction of pseudo-MEG-connectivity matrices

Core to the statistical test applied in this paper is construction of null distributions using pseudo-matrices. For these null distributions to be realistic, the mathematical structure of the pseudo-matrices must mimic effectively the genuine structure of resting state MEG adjacency matrices. To explain this, consider first the example of measuring correlation between time series: If time series are temporally smooth, the effective number of degrees of freedom is reduced. This means a high correlation coefficient may not necessarily imply significant correlation because as smoothness is increased, high correlation is more likely to occur by chance. The same applies to our matrices: the real MEG and fMRI matrices exhibit natural smoothness due to the logical ordering of brain regions in the AAL atlas. If that same spatial smoothness is not maintained in the pseudo-matrices, (for example if pseudo-matrices were generated by random shuffling of the order of AAL regions) correlation coefficients measured between a pseudo-matrix and the fMRI matrix will always be lower. This would lead to a biased null distribution and artefactual assignment of significance. In order to solve this problem we must construct pseudo-matrices with an inherently smooth spatial structure which mimics the genuine MEG derived matrices. To do this we employ a methodology based upon eigenvalue decomposition and phase randomisation. First assume that the original MEG derived adjacency matrix is denoted as **W**_*f*_. Eigenvalue decomposition allows this to be deconstructed such that,

(A1) $$W_{f}=\mathrm{US}U^{T}$$

where **S** is a diagonal matrix whose elements contain the eigenvalues of **W**_*f*_ and the columns of **U** contain the associated eigenvectors, ***u***_*i*_, where 1 ≤ *i* ≤ 78, so that $U=\left[\begin{array}{ccc} u_{1} & \ldots & u_{78} \end{array}\right]$ (recall we have 78 AAL regions). We next manipulate the eigenvectors, ***u***_*i*_, in a meaningful way in order to define a new pseudo-matrix with similar structure to **W**_*f*_. First note that all ***u***_*i*_ are 78 element vectors, with a single element for every region, thus we can write ***u***_*i*_ = ***u***_*i*_(*r*) where *r* represents brain region. Fourier transformation of ***u***_*i*_(*r*) gives:

(A2) $$f_{i}\left(k_{r}\right)=A\left(k_{r}\right)e^{i\emptyset \left(k_{r}\right)}$$

where ***A***(*k*_*r*_) represents the Fourier amplitudes and ∅(*k*_*r*_) represents the Fourier phases. Note that, although the Fourier conjugate dimension, *k*_*r*_, is not physically meaningful, it can be thought of as representing spatial frequency across the 78 ordered AAL brain regions. To manipulate the eigenvectors, we employ an approach used by Prichard and Theiler (1994) based upon phase randomisation. We first construct phase randomised eigenvectors in Fourier space as:

(A3) $$g_{i}\left(k_{r}\right)=A\left(k_{r}\right)e^{i\left(\emptyset \left(k_{r}\right)+\theta \left(k_{r}\right)\right)}$$

where *θ*(*k*_*r*_) contains random numbers sampled from a uniform distribution between *0* and *2π*. Inverse Fourier transform of ***g***_*i*_(*k*_*r*_) gives ***v***_*i*_(*r*), the phase randomised eigenvectors for all 78 AAL regions. It should be noted that phase randomisation in this way maintains the spatial frequencies (hence smoothness across AAL regions) in the eigenvector, but destroys the phase information. Application of this methodology to all eigenvectors allows construction of a new randomised set of eigenvectors so that $V=\left[\begin{array}{ccc} v_{1} & \ldots & v_{78} \end{array}\right]$. The final pseudo-matrix can then be constructed as:

(A4) $$W_{\mathrm{pseudo}}=\mathrm{VS}V^{T}.$$

Multiple realisations of **W**_*pseudo*_ can be generated based upon different realisations of ***θ***(*k*_*r*_).

Fig. A1A shows the effect of phase randomisation on a single eigenvector. The blue trace shows a single eigenvector taken from a genuine MEG (alpha band AEC) adjacency matrix, whilst the green trace shows the phase randomised version. In Fig. A1B, the left hand panel shows the same genuine MEG adjacency matrix whilst the right hand panel shows a phase randomised pseudo-matrix.

As noted above, the mathematical structure (smoothness) in our real MEG and fMRI derived adjacency matrices results from the ordering of the 78 AAL brain regions; regions are ordered by hemisphere (left followed by right) and run approximately anterior to posterior. This logical ordering promotes periodicity in the matrix. For example, the four red squares that are prominent in the left panel of Fig. A1B are generated by genuine visual cortex connectivity. The two squares close to the leading diagonal represent left and right visual cortex connectivity, whereas the off diagonal squares represent interhemispheric connectivity. This structure is only apparent because of the way in which visual regions are clustered together, and the way the left and right hemispheres are split; i.e. if the ordering of the brain regions was randomised, such structure (and therefore the spatial smoothness of the matrix) would be destroyed. In the phase randomisation approach, the periodicity (hence smoothness) in the genuine eigenvector shown in Fig. 1A (blue trace) (which results from the cross hemisphere split in the AAL region ordering) is mimicked in the phase randomised eigenvector (green trace). This is because the amplitudes of the Fourier components of the eigenvector are maintained. However, the precise brain regions involved differ. Application of this approach to all eigenvectors means that the spatial smoothness and periodicity, inherent to the genuine MEG matrices, is also apparent in the pseudo-matrices. However any genuine connectivity information is destroyed making them appropriate for null distribution calculation.
